# Liver cancer mortality trends in South Africa: 1999–2015

**DOI:** 10.1186/s12885-018-4695-9

**Published:** 2018-08-07

**Authors:** Daniel Mak, Mazvita Sengayi, Wenlong C. Chen, Chantal Babb de Villiers, Elvira Singh, Anna Kramvis

**Affiliations:** 1 Hepatitis Virus Diversity Research Unit (HVDRU), Department of Internal Medicine, School of Clinical Medicine, Faculty of Health Sciences, University of Witwatersrand, Johannesburg, South Africa; 2 National Cancer Registry, National Health Laboratory Service, Johannesburg, South Africa; 3 School of Public Health, University of the Witwatersrand, Johannesburg, South Africa; 4 Division of Human Genetics, School of Pathology, Faculty of Health Sciences, University of the Witwatersrand, Johannesburg, South Africa; 5 Sydney Brenner Institute for Molecular Bioscience, University of the Witwatersrand, Johannesburg, South Africa

**Keywords:** Liver cancer, Trends, Mortality, Incidence, South Africa, Sub-Saharan Africa

## Abstract

**Background:**

In South Africa (SA), liver cancer (LC) is a public health problem and information is limited.

**Methods:**

Joinpoint regression analysis was computed for the most recent LC mortality data from Statistics South Africa (StatsSA), by age group, sex and population group. The mortality-to-incidence ratios (MIRs) were calculated as the age-adjusted mortality rate divided by the age-adjusted incidence rate.

**Results:**

From 1999 to 2015, the overall LC mortality significantly decreased in men (− 4.9%) and women (− 2.7%). Overall a significant decrease was noted in black African men aged 20–29 and 40–49 years, and white women older than 60 years but mortality rates increased among 50–59 and 60–69 year old black African men (from 2010/2009–2015) and women (from 2004/2009–2015). The mortality rates increased with age, and were higher among blacks Africans compared to whites in all age groups - with a peak black African-to-white mortality rate ratio of six in men and three in women at ages 30–39 years. The average MIR for black African men and women was 4 and 3.3 respectively, and 2.2 and 1.8 in their white counterparts. Moreover, decreasing LC mortality rates among younger and the increase in rates in older black Africans suggest that the nadir of the disease may be near or may have passed.

**Conclusions:**

Findings of population-age subgroup variations in LC mortality and the number of underdiagnosed cases can inform surveillance efforts, while more extensive investigations of the aetiological risk factors are needed. Impact: There was a large race, sex and age differences in trends of LC mortality in SA. These findings should inform more extensive evaluation of the aetiology and risk factors of LC in the country in order to guide control efforts.

## Background

Liver cancer (LC) is the second largest contributor to cancer mortality worldwide [1], with 810,000 LC deaths recorded in 2015 [2]. The most common type of LC globally is hepatocellular carcinoma (HCC), accounting for 75 to 90% of primary LCs, followed by cholangiocarcinoma [3]. In 2015, close to 20.5 million years of healthy life [disability-adjusted life-years (DALYs)] were lost as a result of this cancer and thus it remains an important public health issue worldwide [2].

In the United States, population group disparities in LC mortality have been reported, with higher rates among African American young adults (35–49 years) and older ages (50–64 years) (2.0 and 18.6/100,000, respectively) compared to Whites (0.9 and 7.7/100,000, respectively) [4]. Invariably, African-to-white American mortality rate ratios are >1 in both men (1.7) and women (1.4) [5]. Declining LC mortality rates have been reported in young African and white Americans (5.3 and 2.8% annually, respectively) while those of older ages are increasing (by 6.2 and 6.3% annually, respectively). In America, Africans are more frequently diagnosed with LC at a younger age than Whites [6]. The former have larger tumour size, more advanced tumour stage/with metastatic disease, lower levels of alpha-fetoprotein and are least likely to present with cirrhosis [7, 8]. Reasons for these disparities have been attributed to differences in the prevalence of major risk factors including hepatitis B and/or C virus (HBV/HCV), obesity and diabetes [9], and to some degree, because of barriers to accessing high-quality care [10].

In contrast, data on LC mortality in South Africa (SA), and most regions of sub-Saharan Africa, are limited. The International Agency for Research on Cancer GLOBOCAN estimated 37,353 LC deaths occurred in this subcontinent in 2012 and predicted this number to increase to 64,525 by 2030 [1]. Thus, the present study aims to delineate the patterns and temporal trends of LC mortality in SA, based on currently available data from the national death registry from 1999 to 2015 by sex, age and population group.

## Methods

### Data sources and selection criteria

Cause of death was based on death certificate information reported to Statistics South Africa (StatsSA), a national statistical service that compiles routine mortality statistics based on medical certification of the cause of death registered with the Department of Home Affairs (DHA). LC (C22.0), as the first or underlying cause of death was selected according the ICD-10 for the period of 1999 to 2015. Analyses were restricted to four large population groups that were defined according to the SA National Census as Black Africans, White, Coloured (mixed ancestry) and Indian/Asian [11]. In 2015, each population group accounted for 80.5, 8.3, 8.8 and 2.5% of the SA population, respectively [12]. Since the majority of cases reported each year for the Coloured and Indian/Asian populations were fewer than 100, both these groups were excluded from further analysis.

### Statistical analysis

Age-standardized incidence rates (ASIRs) and mortality rates (ASMRs) per 100,000 persons were calculated using mid-year population estimates provided by StatsSA and by direct standardization with the Segi’s World Standard Population (1960) [13], by population group, sex and age group (summarized into 10-year age groups: 20–29, 30–39, 40–49, 50–59, 60–69 and 70+ years). Joinpoint regression trends were examined by population group, sex and age groups, based on a two joinpoint segment model [14]. Based on the criteria used in the National Cancer Institute’s Cancer Trends Progress Report [15] and modified in a published report by Altekruse et al. [4], the trends were described as annual percentage change (APC) and categorized into five groups: 1: Significant decrease (APC < 0%, *p* < 0.05); 2: Non-significant decrease (APC < − 0.5%, *p* > 0.05); 3: Stable (Absolute value of rate change less than or equal to 0.5% per year, *p* > 0.05); 4: Non-significant increase (APC > + 0.5% per year, *p* > 0.05); and 5: Significant increase (APC > 0%, *p* < 0.05). The APC and average annual percentage change (AAPC) were calculated using Joinpoint software (Version 4.5.0.1) from the Surveillance Research Program of the US National Cancer Institute [16]. Statistical significance was taken at *p* ≤ 0.05. The mortality-to-incidence ratios (MIRs) were calculated as the age-adjusted mortality rate divided by the age-adjusted incidence rate for LC from 1999 to 2012, and was used to compare population group and sex disparities. Age-Adjusted incidence rates for MI ratios were derived from the South African National Cancer Registry’s pathology-based cancer surveillance system.

## Results

**Fig. 1:**
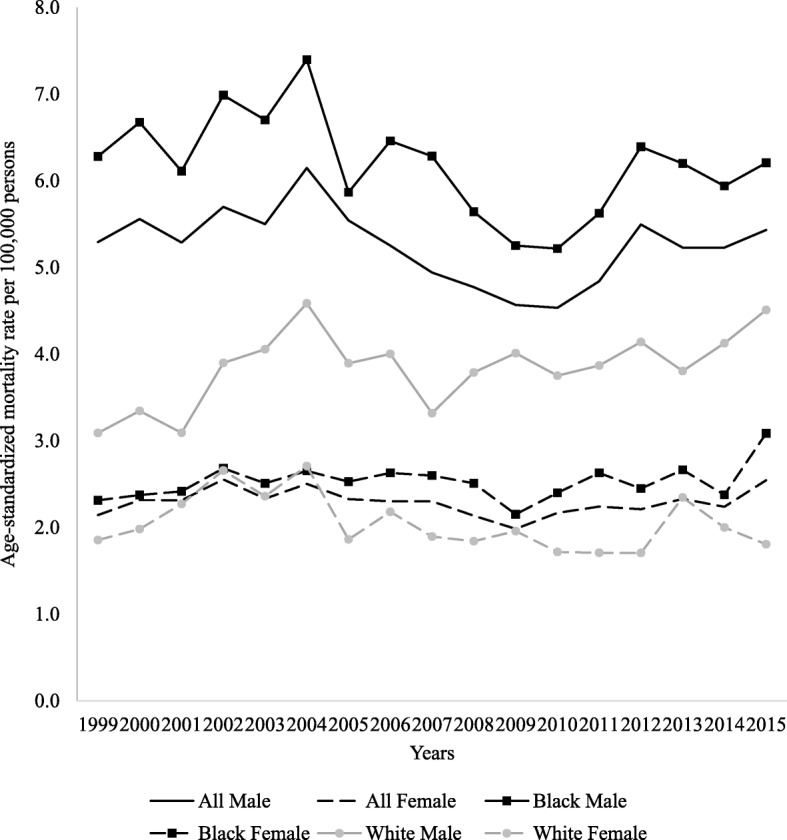
Liver cancer age-standardized mortality rates in South Africa by population group and sex, 1999–2015

Figure 1 shows the sex and population group-specific overall ASMR for LC in SA. The results of the joinpoint regression analysis, the APCs for each trend, and the AAPCs in both genders are depicted in Table 1. During the 17-year mortality study period, a total of 27,791 deaths from LC were reported. Of the total cases of known population group (22,435, 81%), majority were men (62.1%) and black African (73.2% vs 15.4% whites). Women died of LC at significantly older ages than men, with 83.5% versus 79.1% being older than 40 years, and 59.6% versus 50.1% being older than 60 years, respectively. LC ASMRs in men and women (overall) did not significantly change throughout the study period (5.3 and 2.1/100,000 in 1999 to 5.4 and 2.5/100,000 in 2015), with an AAPC of 0.3 and 0.8% (*p* > 0.05) respectively. The segmented joinpoint analysis identified a period of decrease in mortality rates (men: 2004–2009, APC of − 4.9%; women: 2002–2009, APC of − 2.7%), after which the rates began to increase (2009–2015, APCs: 3.4 and 2.6% respectively). In men, age-group analysis revealed that, during the entire study period, the mortality rates decreased significantly for all age groups >20 years old; whereas in women this only included 40–49 and >60 year olds. However, segmented joinpoint analysis by age groups distinguished between two different time periods, i.e. an initial period characterized by a significant decrease in 50–59 year old men (1999–2010) and 60–69 year old women (1999–2009), and a second period with an increase or levelling-off in rates until 2015.

**Table 1:** Joinpoint analysis of age-standardised liver cancer mortality rates by sex and age group in South Africa; 1999–2015

All		Trend 1		Trend 2		Trend 3	
Age (Years)	AAPC (95% CI)	Range of Years	APC	Range of Years	APC	Range of Years	APC
Men							
Overall	0.3 (− 1.2, 1.8)	1999–2004	2.1 (− 0.7, 5)	2004–2009	−4.9* (− 8.6, − 1.1)	2009–2015	3.4* (1.2, 5.6)
20–29	−3.2* (− 4.9, − 1.4)	1999–2015	−3.2* (− 4.9, − 1.4)				
30–39	−1.8* (− 3, − 0.6)	1999–2015	−1.8* (− 3, − 0.6)				
40–49	−2.1* (− 3.2, − 1.1)	1999–2015	−2.1* (− 3.2, − 1.1)				
50–59	−1.8* (− 3.3, − 0.3)	1999–2010	− 3.7* (− 5, − 2.4)	2010–2015	2.6 (− 2, 7.3)		
60–69	−3.4* (− 4.4, − 2.4)	1999–2015	−3.4* (− 4.4, − 2.4)				
70+	− 2.3* (− 3.3, − 1.3)	1999–2015	−2.3* (− 3.3, − 1.3)				
Women							
Overall	0.8 (−0.6, 2.2)	1999–2002	5.6 (−0.6, 12.2)	2002–2009	−2.7* (− 4.7, − 0.7)	2009–2015	2.6* (0.6, 4.8)
20–29	−1.5 (− 3.1, 0.1)	1999–2015	−1.5 (− 3.1, 0.1)				
30–39	−1.4 (−3, 0.3)	1999–2015	− 1.4 (− 3, 0.3)				
40–49	−1.7* (− 2.4, − 1)	1999–2015	−1.7* (− 2.4, − 1)				
50–59	−0.9 (−1.9, 0)	1999–2015	− 0.9 (− 1.9, 0)				
60–69	−2.6* (− 3.9, − 1.3)	1999–2009	− 4.4* (− 5.8, − 3)	2009–2015	0.5 (− 2.7, 3.7)		
70+	− 2.3* (− 3, − 1.5)	1999–2015	−2.3* (− 3, − 1.5)				

*The average annual percentage change (AAPC) and/or annual percent change (APC) is statistically significant (*p* < 0.05)

**Fig. 2:**
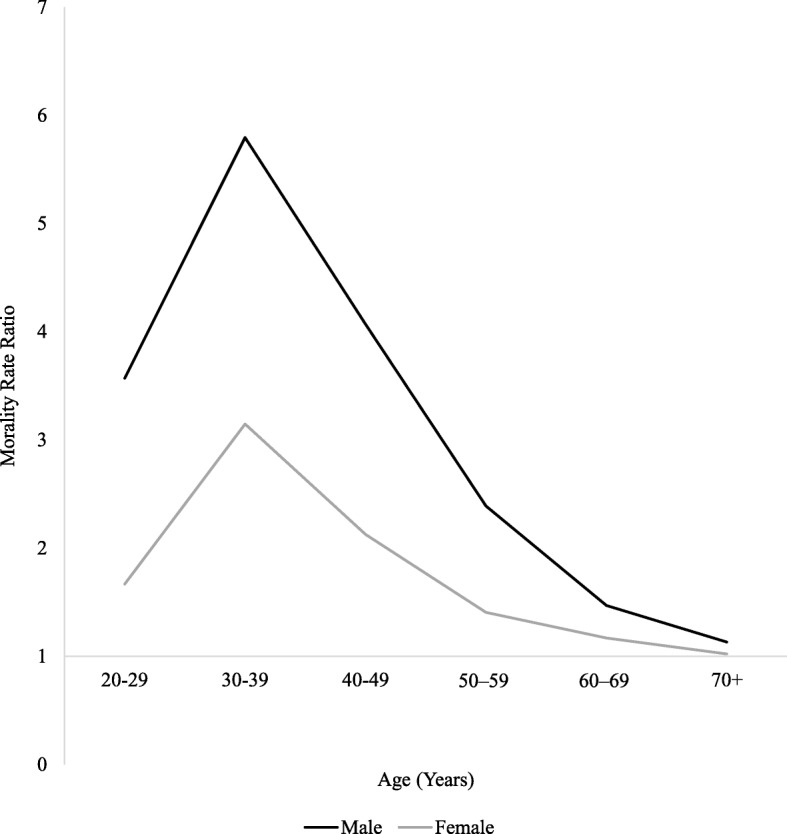
Black African-to-white liver cancer mortality rate ratio in men and women, 1999–2015

**Fig. 3:**
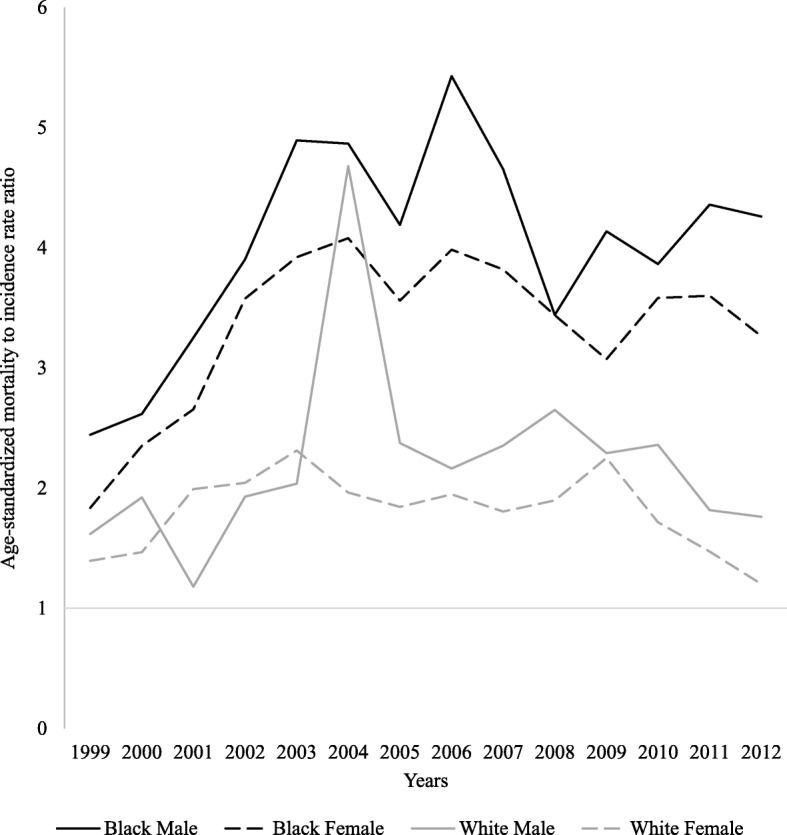
Liver cancer mortality-incidence rate ratio by population group and sex, 1999–2012

Over the study period, population group analysis showed that black African men (6.2/100,000) had the highest LC ASMR, followed by white men (4.5/100,000), black African women (3.1/100,000) and white women (1.8/100,000) (Fig. 1). The corresponding black African-to-white LC mortality rate ratio increased and peaked at ages 30–39 years (6-fold in men and 3-fold in women), but decreased thereafter to around 1.0 in those >70 years old in both sexes (Fig. 2). Segmented joinpoint analyses of population-age subgroups by sex are shown in Table 2. In black Africans, a decreasing or stable trend in ASMRs was observed among younger (<50 year old) and (>70 year) old men and women from 1999 through 2015. Interestingly, joinpoint analysis identified two different periods for older black Africans (50–59 and 60–69 year old). After an initial period of decreasing ASMRs, a marked non-significant increase was found thereafter in men (5.4 and 2.5% respectively, *p* > 0.05), but was significant in women (2.6 and 6.3% respectively, *p* < 0.05). In whites, age-group analysis revealed that, throughout the entire study period, ASMRs either remained stable (men: 40–69 years; women: 40–49 years) or decreased (men: >70 years; women: >50 years). For both the black African and white populations, mortality rates were higher than incidence rates reported to the South African National Cancer Registry, yielding an average MIR of 4 and 3.3 in black African men and women, and 2.2 and 1.8 white men and women (Fig. 3).

**Table 2:** Age-adjusted liver cancer mortality rates joinpoint trends by population group, sex and age group in South Africa; 1999–2015

Direction	Sex	Population Group	Age, Years	Trend 1		Trend 2	
				Period	APC (95% CI)	Period	APC (95% CI)
1. Significant Decrease							
	Men						
		Black	20–29	1999–2015	− 2.3* (− 4.2, − 0.4)		
		Black	40–49	1999–2015	− 1.1* (− 2.2, 0)		
	Women						
		White	60–69	1999–2015	−2.6* (− 4.9, − 0.3)		
		White	70+	1999–2015	−3.5* (− 4.9, − 2.1)		
2. Non-significant Decrease							
	Men	Black	70+	1999–2015	−1.3 (− 2.5, 0)		
		White	70+	1999–2015	−0.7 (− 2.2, 0.9)		
	Women						
		Black	20–29	1999–2015	−1.5 (−4.2, 1.4)		
		Black	40–49	1999–2015	−0.9 (− 2, 0.1)		
		White	50–59	1999–2015	−0.9 (−3.8, 2.1)		
3. Stable							
	Men						
		Black	30–39	1999–2015	−0.2 (−1.5, 1.1)		
		White	40–49	1999–2015	−0.5 (−4.5, 3.6)		
		White	50–59	1999–2015	−0.1 (−2.6, 2.5)		
		White	60–69	1999–2015	0.4 (−1.2, 2)		
	Women						
		Black	30–39	1999–2015	−0.2 (−1.9, 1.6)		
		Black	70+	1999–2015	−0.1 (−1.2, 1)		
		White	40–49	1999–2015	−0.2 (−8.9, 9.2)		
4. Non-significant Increase							
	Men						
		Black	50–59	1999–2010	−3.7* (−5.3, −2.2)	2010–2015	5.4 (− 0.1, 11.2)
		Black	60–69	1999–2009	−5.2* (−7.3, − 3)	2009–2015	2.5 (− 2.4, 7.6)
5. Significant Increase							
	Women						
		Black	50–59	1999–2004	−5.2 (− 10.4, 0.4)	2004–2015	2.6* (0.8, 4.4)
		Black	60–69	1999–2009	−4.9* (− 6.9, − 2.8)	2009–2015	6.3* (1.5, 11.3)

*The annual percent change (APC) is statistically significant (*p* < 0.05)

## Discussion

The data-set used (from StatsSA) is considered to provide the most comprehensive and representative information on mortality patterns in the country. Our analysis highlights an overall sociodemographic shift in LC mortality in SA over the past two decades. More specifically, there was a shift from a decreasing to an increasing trend in LC mortality among older black Africans (50–69 years). In contrast, mortality trends were stable and decreasing in the white population of the same sex and age groups. Black Africans were more likely to die from LC compared to whites. These racial differences in LC mortality were highest in the 30–39 year old age group with black African-to-white mortality ratios of six in men and three in women. For both the black African and white populations, mortality rates were higher than incidence, yielding an average MIRs of 4 and 3.3 in men and women in the former, and 2.2 and 1.8 in the latter population. Data of most individuals with LC-related deaths were not captured in the South African National Cancer Registry’s pathology-based cancer registry. To the best of our knowledge, this is the first such analysis of time trends that demonstrated a LC mortality differential between the black African and white populations by sex nationwide.

In SA, accounting for population-age subgroup disparities in LC mortality is likely multifaceted and may be a consequence of socio-cultural, biological and socioeconomic factors. While the establishment of policies in the mid-1990s dealing with better quality and more accessible health care services may have improved overall patient survival over time [17], and ultimately decreasing mortality as a consequence of increased utilization of these services and the better application of early detection strategies, other factors may impede the ability to seek and obtain timely services [18]. For example, evidence from earlier studies in SA show that the bottom segment of the distributions in income [19], education/literacy [20], dependence on public health care programs [21] are dominated by black Africans and health insurance coverage shares are far from proportionate with population shares (73.3% and 10.6% of white and black Africans with health insurance in 2015, respectively) [20]. In a study in SA, the authors reported that of the 22 patients with HCC who received surgical treatment, the surgical rates were lower for black Africans [22]. While it is possible that cultural beliefs and attitudes may have impacted on the appropriate treatment decision-making [23], other reports have attributed this disparity to differences in the biology and presentation of the disease [24–26]. Previous studies have suggested that black Africans are more likely to be diagnosed symptomatically in the absence of any screening methods, at a later stage of disease with larger tumours (80–90%) [24, 25], and when curative treatments are no longer feasible [26].

Alternatively, changes in the prevalence of risk factors may play a role. For example it is well recognized that HBV is a primary risk factor and its prevalence differs among black Africans (4–16%) and whites (0.2%) in the same manner as does the burden of LC in SA [27, 28]. While the vaccine against HBV has been shown to be efficacious at reducing HBV infections, and ultimately HCC, among vaccinated cohorts of children [29, 30], they remain ineffective for most chronically infected adults born prior to its incorporation into the Expanded Programme on Immunisation in 1995. Comparisons of a study in the 1990s [31] to that of the 2000s [32] showed that HBV-infected HCC cases are generally diagnosed in their 30s, and the HBsAg rates in HCC patients have increased in men (38.2% versus 43.2%, respectively) and women (24.1% versus 35.9%, respectively). In contrast, HCV infection is relatively uncommon in SA (0.75% and 0.16% in black Africans and whites, respectively) [33], but affects older patients who are in their 50s [31]. Unlike HBV infection, HCV-positivity in HCC patients decreased (28.7% versus 5.4%) from the 1990s [31] to 2000s [32].

Data on other potential factors associated with lifestyle behaviours (obesity, diabetes, alcohol consumption and dietary exposure to aflatoxin) influencing LC rates are limited in SA, with our recent study showing minimal effects [32]. Thus it is difficult to determine which of these factors predominantly influenced the trends in LC burden, because the population attributable fraction of these factors is not as large as hepatitis infection. Future studies should explore the impact of these aetiological factors on LC mortality and incidence at an individual level.

In SA, it is clear that human immuno-deficiency virus (HIV) epidemic has had a major demographic and health impact particularly in black African population. Findings from previous studies from SA and Uganda that have failed to show increases in HCC risk among HIV-infected persons through the pre-ART years (pre-2004 in SA), possibly as a result of the competing risks of AIDS-related deaths [34, 35]. However, an increase in LC cases has been noted in the ART era in developed countries [36]. Our results show an upswing in LC mortality rates in black Africans during the inception of ART in 2004 [37] (number of adults accessing ART was 4.9%) [38] and the massively scaled up program in 2009 (61%) [39]. In Uganda, a study reported that with each 10% increase in ART coverage, LC incidence increased by 12% [40]. It has been suggested that the change in the spectrum of liver disease is shifting from opportunistic infections to sequelae of chronic HBV/HCV infections, medication toxicities, alcoholism, and fatty liver [41], possibly as a result of increased longevity. Although these associations need to be further investigated in SA, it is conceivable that results presented here may be interpreted as revealing an effect of ART given that both the number of people in SA living with HIV, and those with access to ART are among the highest in the world [42]. Findings from this study raise considerable concern that the burden of LC may expand concurrently with the implementation of an universal ART eligibility to all 7.1 million HIV-positive South Africans from 2016 [43], further highlighting the need for a comprehensive program focused on population-based cancer surveillance and control including LC in SA [44].

In a region characterized by the poor surveillance systems, tracking the emerging trends in the death rates is an essential tool to assist with cancer-control planning, early detection, and prevention efforts. Additionally, because preventing deaths must be an important part of any competent cancer prevention and control effort, the statistics described in this study also address an important need – to describe and highlight the knowledge gap of LC mortality in relation to incidence. The MIRs of LC in men and women in our study (black African: 4 and 3.3; white: 2.2 and 1.8, respectively) was significantly great compared that reported in another HBV endemic region, China (0.91 and 0.92, respectively) [45]. Given the high MIR ratios reported in our study, pathology-based cancer registration is inadequate to characterise LC epidemiology in SA. The establishment of a nationally representative population-based cancer registration should be a priority for cancer control in SA as it will capture cases of LC diagnosed clinically and radiologically in addition to laboratory based diagnoses.

Several potential limitations should be acknowledged. There have been considerable improvements in the national coverage and completeness of death registration, but shortcomings of the quality of death certification have been suggested in ill-defined and misattributed causes of single-cause data [46]. Considering that the liver is a frequent site of cancer metastasis, cause of death determination using death certificates may result in false-positives in the absence of biopsy [47]. Additionally, failure or under-ascertainment of cause-of-death could lead to bias in death registration, particularly in resource-limited regions [39]. Despite regular census data being available, concerns about the accuracy of earlier population estimates may suggest the possible introduction of uncertainty in estimated mortality rates [12]. The number of LC-related deaths may be overestimated because StatsSA collects and processes death information irrespective of the deceased’s citizenship. The livelihood conditions in SA make it an attractive destination for migration and therefore cross border migrations are frequent [48]. On the other hand, the inability of the SA health-system to meet the health needs of the migrants means that when their health deteriorates they return to their place of origin to seek healthcare and support [49]. Trends analyses on LC rates in the current study were descriptive analyses at population levels without inference at individual levels. Because data at individual level on socioeconomic status and various potential confounders were not available, interpretations from the results do not necessarily hold true for individuals.

## Conclusion

In summary, this is the first study to estimate the total burden of liver cancer deaths in SA. Our results indicate a rising trend for this disease in middle to older aged black African men and women in the recent decade. The reasons for this are likely manifold, but may be a consequence of improved life expectancy and rising prevalence of risk factors related to lifestyle behaviours. Further research into the aetiologic factors contributing to population-age sub-group mortality patterns are needed, to provide informative interventions to curb the rising burden of liver cancer deaths and to reduce the population group disparities. Our findings may be used to inform national health and social development policies in SA and other regions of southern Africa.

## Abbreviations

AAPC: Average annual percentage change
APC: Annual percentage change
ASIR: Age-standardized incidence rate
ASMR: Age-standardized mortality rate
HBV: Hepatitis B virus
HCC: Hepatocellular carcinoma
HCV: Hepatitis C virus
HIV: Human immuno-deficiency virus
LC: Liver Cancer
MIR: Mortality-to-incidence ratio
SA: South Africa
StatsSA: Statistics South Africa
